# Stabilization by Configurational Entropy of the Cu(II) Active Site during CO Oxidation on Mg_0.2_Co_0.2_Ni_0.2_Cu_0.2_Zn_0.2_O

**DOI:** 10.1021/acs.jpclett.0c00602

**Published:** 2020-04-20

**Authors:** Martina Fracchia, Paolo Ghigna, Tommaso Pozzi, Umberto Anselmi Tamburini, Valentina Colombo, Luca Braglia, Piero Torelli

**Affiliations:** † Dipartimento di Chimica, Università di Pavia, V. le Taramelli 13, I-27100, Pavia, Italy; ‡ INSTM, Consorzio Interuniversitario per la Scienza e Tecnologia dei Materiali, Via Giusti 9, I-50121 Firenze, Italy; § Dipartimento di Chimica, Università degli Studi di Milano, Via Golgi 19, I-20133 Milano, Italy; ∥ CNR - Istituto Officina dei Materiali, TASC, I-34149 Trieste, Italy

## Abstract

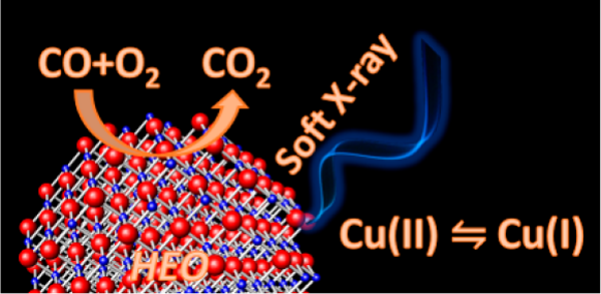

The mechanisms of CO oxidation on the Mg_0.2_Co_0.2_Ni_0.2_Cu_0.2_Zn_0.2_O high-entropy oxide were studied by means of operando soft X-ray absorption spectroscopy. We found that Cu is the active metal and that Cu(II) can be rapidly reduced to Cu(I) by CO when the temperature is higher than 130 °C. Co and Ni do not have any role in this respect. The Cu(II) oxidation state can be easily but slowly recovered by treatment of the sample with O_2_ at ca. 250 °C. However, it should be noted that CuO is readily and irreversibly reduced to Cu(I) when it is treated with CO at *T* > 100 °C. Thus, the main conclusion of this work is that the high configurational entropy of Mg_0.2_Co_0.2_Ni_0.2_Cu_0.2_Zn_0.2_O stabilizes the rock-salt structure and permits the oxidation/reduction of Cu to be reversible, thus permitting the catalytic cycle to take place.

Low-temperature CO oxidation, perhaps the most extensively studied reaction in the history of heterogeneous catalysis, is becoming increasingly important in the context of cleaning air and lowering automotive emissions.^1^ Hopcalite catalysts (manganese and copper spinel) were originally developed to purify air in submarines, but they are not particularly active at ambient temperatures and are also deactivated by the presence of moisture.^2^ Noble metal catalysts, on the other hand, are water-tolerant but usually require temperatures above 100 °C for efficient operation.^3^ Gold exhibits high activity at low temperatures and superior stability under moisture, but only when deposited as nanoparticles on transition-metal oxides.^4^ The development of active and stable catalysts without noble metals for low-temperature CO oxidation under ambient atmosphere remains a significant challenge. Among the metal oxides, Co_3_O_4_ spinel is the most active for CO oxidation,^5^ but it is severely deactivated by trace amounts of moisture (about 3–10 ppm) that are usually present in the feed gas. In fact, under dry conditions with a moisture content below 1 ppm, which can be obtained by passing the reaction gas through molecular-sieve traps cooled to dry ice temperature, Co_3_O_4_ is intrinsically active for CO oxidation^6^ even below a temperature of −54 °C. However, in normal feed gas, most of the active sites of Co_3_O_4_ are covered by H_2_O, so the adsorption of CO and oxygen is appreciably hindered. Alumina-supported Co_3_O_4_ was reported to give 50% CO conversion at −63 °C for a normal feed gas, but the CO conversion was obtained with a transient method^7^ rather than at steady state. Mechanistic studies show that CO molecules interact preferably with the surface Co^3+^ cation, which is the only favorable site for CO adsorption, as confirmed both theoretically^8^ and experimentally.^9^ The oxidation of the adsorbed CO then occurs by extraction of the surface oxygen that might be coordinated with three Co^3+^ cations. This rationale suggests that the presence of transition metals in high oxidation states is a prerequisite for finding effective catalysts for the CO oxidation reaction; indeed, all of the oxide catalysts for the CO oxidation reaction do have metals in high or mixed oxidation or valence states. In this context, the recent discovery of a noticeable catalytic activity of the high-entropy oxide (HEO) Mg_0.2_Co_0.2_Ni_0.2_Cu_0.2_Zn_0.2_O, which has a rock-salt structure, toward the CO oxidation reaction^10^ is quite surprising, as all the cations are formally in the M(II) oxidation state. HEOs are a recently discovered class of materials^11^ where a particular crystal structure, which in general is different from that of the parent compounds, is stabilized in a multicomponent system (generally, five components or more) by the large amount of configurational entropy.^12^ The main driver for the growing interest in HEOs is the potential to obtain novel properties by exploiting the enormous number of possible elemental combinations; in addition, the synthesis of these materials is facile, and several synthetic routes can be explored for obtaining highly reproducible materials.

As previously mentioned, the catalytic activity of the Mg_0.2_Co_0.2_Ni_0.2_Cu_0.2_Zn_0.2_O HEO at quite low temperatures (250–300 °C) toward the CO oxidation reaction poses a series of questions concerning the mechanisms of the catalytic circle, as none of the parent oxides has such a reactivity. The questions mainly concern the local electronic structure of the transition metals (Co, Ni, Cu, and Zn), their oxidation states, the nature of the active surface site, and possible changes in all of these properties during the reaction course. Here we plan to tackle this problem by operando soft X-ray absorption spectroscopy (soft-XAS) experiments at the L_2,3_ edges of the transition metals (TMs). In recent years, in situ and operando investigations at L edges of transition metals have received increasing attention in the field of catalysis.^13−15^ In fact, soft-XAS in total electron yield (TEY) mode combines two unique features: (i) the capability to directly monitor the density of empty 3d states of TMs when the L_2,3_ edges are selected and (ii) the surface sensitivity, which because of the low value of the electron escape depth limits the thickness of the probed sample to a few atomic layers below the surface. More details on the choice of soft-XAS as a mechanistic tool for this catalytic reaction can be found in the Supporting Information.

Figure 1 shows the Cu L_2,3_-edge XAS spectra of the HEO under different conditions. Spectra of CuO and Cu_2_O are also shown for a better reference. The spectrum of CuO presents a clear, intense peak at both the L_3_ and L_2_ edges that is due to electronic transitions from 2p states to the empty 3d states of the d^9^ electronic configuration of Cu(II).^16^ The interpretation of the Cu_2_O spectrum is more complex, as Cu(I) is formally in the d^10^ electronic configuration and therefore the 2p → 3d electronic transitions would in principle be impossible. However, there is a general consensus that Cu(I) in linear coordination in Cu_2_O gives rise to an unusually large partial 3d character in the empty density of states.^16,17^ In any case, it is clear that while the peak at the L_3_ edge at ca. 931.3 eV is attributed to Cu(II), the peak at the L_3_ edge at ca. 934.8 eV is a clear signature of Cu(I). We can now discuss the Cu L_2,3_-edge spectra of the Mg_0.2_Co_0.2_Ni_0.2_Cu_0.2_Zn_0.2_O HEO. At room temperature and in inert gas, the spectrum bears a close resemblance to that of CuO; this is reasonable because in the HEO with the rock-salt structure, Cu(II) has an octahedral environment similar to that in CuO.^11^ It should also be noted that the Cu L_2,3_-edge spectrum can be properly reproduced by multiplet calculations using an undistorted octahedral Cu(II) model with a d^9^ configuration (see Figure S1 in the Supporting Information).

**Figure 1:**
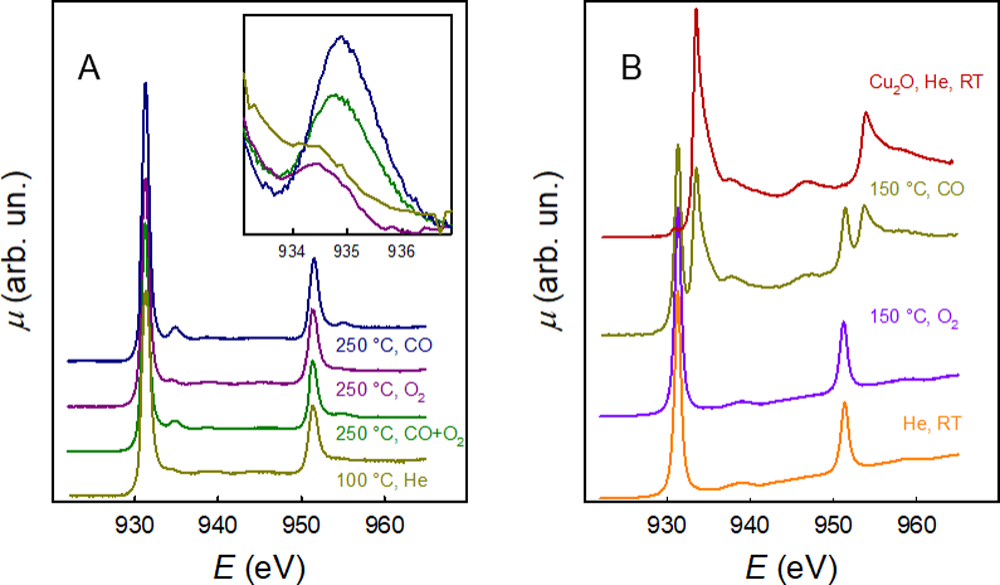
(A) Cu L_2,3_-edge XAS spectra of the Mg_0.2_Co_0.2_Ni_0.2_Cu_0.2_Zn_0.2_O HEO material under different conditions. The inset shows on an enlarged scale the Cu(I) peak at ca. 934.8 eV. (B) Cu L_2,3_-edge XAS spectra of CuO under different conditions and Cu_2_O at room temperature. In this panel, non-normalized spectra are shown.

When the sample is heated at ca. 250 °C in the stoichiometric CO + ^1^/_2_O_2_ gas mixture, a peak at ca. 934.8 eV starts to appear, as is apparent in the green curve in Figure 1; according to the above discussion, this is the signature of Cu(I). At this temperature, the CO_2_ gas sensor shows that the CO oxidation has reached the maximum rate (see Figure S2). The Cu(I) peak amplitude can be reduced by stopping the CO flow and flowing only oxygen on the sample (dark-pink line in Figure 1). This result shows unequivocally that CO oxidation on the HEO proceeds via adsorption of CO on the Cu sites at the surface. This adsorption causes a charge transfer from CO to Cu, thus leading to Cu(I). Then, if the temperature is high enough to allow the oxidation of adsorbed CO by O_2_, CO_2_ leaves the surface and some Cu(I) is reoxidized to Cu(II). The finding that some Cu(I) is present when the oxidation reaction takes place is consistent with the fact that the reduction is faster than the oxidation. It should be noted that the reduction/oxidation of Cu takes place at ca. 130 °C, which is well below the temperature at which the CO oxidation rate, as measured by the CO_2_ sensor, begins to be significant (see Figure S3). This may be attributed to the fact that additional activation energy is required for the oxidation of the adsorbed CO. The fractions of Cu(I) at 250 °C in the CO + ^1^/_2_O_2_ gas mixture and in CO can be estimated to be 3 and 8%, respectively (see Figure S4 and Table S1 for further details). Charge compensation of the Cu_Cu_^′^ defects that are created by Cu(II) reduction can be achieved by formation of oxygen vacancies.

Ni and Co, the two other metals of the system that are not in a closed-shell electronic configuration, act as spectators. We cannot detect any change at the Ni and Co L_2,3_ edges, as shown in Figure 2, which displays the Ni and Co L_2,3_-edge XAS spectra under conditions similar to those at the Cu L_2,3_ edge shown in Figure 1. It is well evident that no changes are detected; moreover, the spectra show a very close resemblance to the Ni L_2,3_-edge spectrum of NiO^18^ and the Co L_2,3_-edge spectrum of CoO.^19^

**Figure 2:**
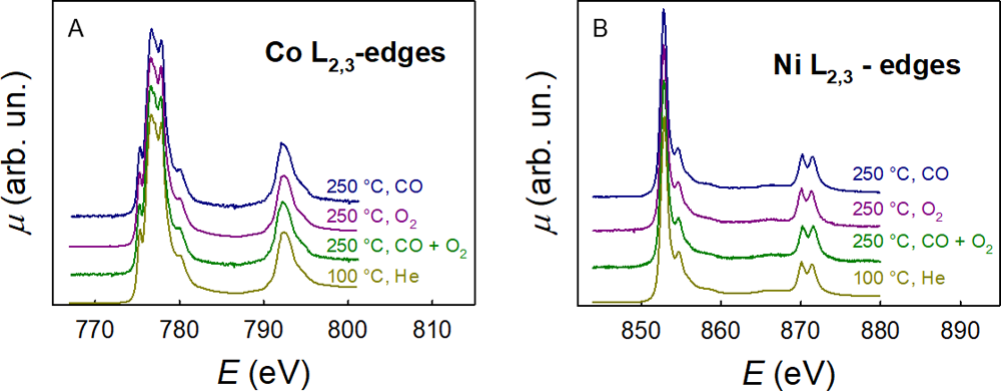
(A) Co and (B) Ni L_2,3_-edge XAS spectra of the Mg_0.2_Co_0.2_Ni_0.2_Cu_0.2_Zn_0.2_O HEO material under different conditions.

As for the spectra at the Cu L_2,3_ edge, the similarity to the corresponding M(II) oxides is due to the fact that in the HEO the transition metals are in an octahedral environment and in the M(II) oxidation state. Also in this case, the spectra are well-interpreted by multiplet calculations using an undistorted octahedral M(II) model (see Figure S1).

The HEO is quite stable toward reduction. In fact, when it is heated at ca. 250 °C in CO (blue line in Figure 1) the intensity of the Cu(I) peak at ca. 934.8 eV increases. However, we should remark that pure copper oxide, CuO, is heavily reduced to Cu(I) when treated in flowing CO at temperatures as low as 150 °C, as shown in Figure 1B. This evidence is important, as it emphasizes the role of the configurational entropy of the HEO material in stabilizing the Cu(II) oxidation state. This fact is indeed very notable, as it may open the way to the tailoring of new catalytic materials by stabilization of unstable oxidation states via the configurational entropy concept. To further investigate this fact, we heated the HEO sample at 235 °C in O_2_ and then switched the flowing gas to CO, keeping the sample at the same temperature for 1 h. The results are shown in Figure 3.

**Figure 3:**
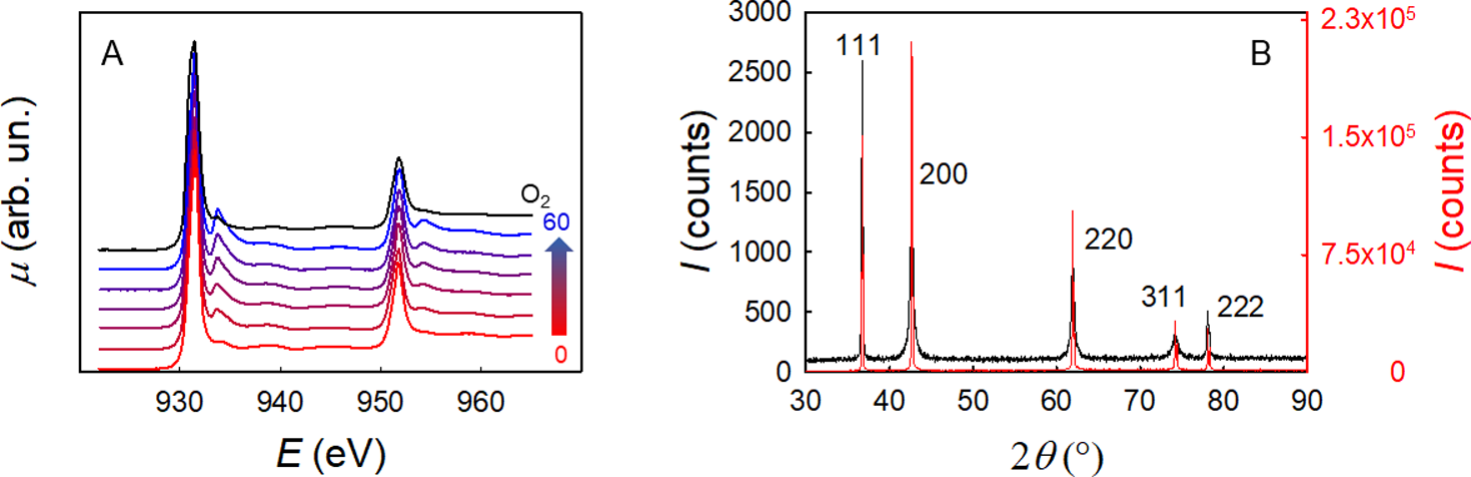
(A) Cu L_2,3_-edge XAS spectra of the Mg_0.2_Co_0.2_Ni_0.2_Cu_0.2_Zn_0.2_O HEO material for different time periods in CO at 235 °C (red to blue lines; numbers 0 → 60 are the dwell times under these conditions expressed in minutes) and then in O_2_ at the same temperature (black line). (B) Comparison of the PXRD patterns of the raw HEO material (red line) and the same material after all of the thermal treatments described in this work (black line). The patterns are indexed according to the rock-salt structure (*Fm*3̅*m*, *a* = 4.2366(5) Å).

The spectra show an increasing intensity of the Cu(I) peak at ca. 934.8 eV with increasing time in CO. After 1 h, the gas flow was switched back to O_2_: this led to a considerable reduction of the intensity of the Cu(I) peak at ca. 934.8 eV, adding further confirmation of the role of configurational entropy in stabilizing the Cu(II)/Cu(I) redox couple. It should be noted that the effects of these thermal treatments on the HEO structure are nontrivial. This is illustrated in Figure 3B, where the powder X-ray diffraction (PXRD) pattern of the as-prepared HEO is compared with that of the material taken out of the soft-XAS operando cell. It is well apparent that while the overall rock-salt structure is preserved, as confirmed by the absence of any additional diffraction effects, all of the reflections except the 111 family display a considerable broadening after the thermal treatments.

This is somewhat in agreement with the role of copper ions in HEO rock-salt samples, which has been demonstrated to unambiguously promote the structural evolution from an *ideal* rock-salt structure to a *distorted* one in copper-containing samples versus copper-free ones.^20^ Rietveld analysis performed on the as-synthesized sample showed that all of the Bragg peaks are indeed indexed in the rock-salt *Fm*3̅*m* space group and that their relative intensities match well with a random distribution of the cations for the *ideal* rock-salt structure of Mg_0.2_Co_0.2_Ni_0.2_Cu_0.2_Zn_0.2_O composition (see the Supporting Information). After the thermal treatments and the change in the oxidation state of copper ions, the broadening of the (200)_c_, (220)_c_, and (311)_c_ peaks nicely corresponds to a tetragonal distortion to the nonisomorphic subgroup *I*4/*mmm* (*a* = 2.9919(2) Å; *c* = 4.2520(4) Å). However, the concomitant presence of a lattice deformation (i.e., loss of long-range ordering, perpendicular to the (111)_c_ direction) cannot be excluded. The details of this phenomenon are currently under investigation by our group to obtain a deeper understanding of the observed peak broadening. Indeed, an EXAFS study of this material revealed a considerable local distortion of the Cu–O octahedron, probably driven by Jahn–Teller distortion around the Cu(II) in the d^9^ electronic configuration.^21^ On the other hand, also Co(II) with the d^7^ configuration is a Jahn–Teller cation, and in addition, Zn is known to preferentially assume the tetrahedral coordination with oxygen with respect to octahedral. Finally, in the HEO structure, each of the metal–oxygen distances is forced by the crystal symmetry to be different with respect to that implied by considering the local environment only. We can therefore speculate that in the HEO structure, several “distortion fields” are present around each of the cations, and the final crystal symmetry is the result of a perfect cancellation of these fields. Removing or altering one of these fields, for example by changing the oxidation state of Cu, and therefore changing the electronic configuration from d^9^ to d^10^, and then removing the Jahn–Teller distortion, would result in a net distortion of the whole crystal.

In summary, in this work we investigated the mechanisms of CO oxidation on the Mg_0.2_Co_0.2_Ni_0.2_Cu_0.2_Zn_0.2_O high-entropy oxide with the rock-salt structure. We found that the only metal involved in the reaction is Cu, while Ni and Co act as spectators. Cu(II) is reduced to Cu(I) by the reactive adsorption of CO. Oxygen can then oxidize the adsorbed CO, forming CO_2_ and recovering the Cu(II) oxidation state. The rock-salt structure of the HEO may therefore have a crucial role in stabilizing the Cu(II)/Cu(I) redox couple. On the other hand, the most thermodynamically stable polymorph of CuO shows a monoclinic structure, different from the cubic rock-salt structure. Stabilization of rock-salt CuO therefore requires additional terms in the Gibbs free energy. Rock-salt CuO can indeed be prepared in the form of nanoparticles, which in turn showing a lower reactivity toward reducing gases compared with the monoclinic polymorph.^22^ For nanoparticles, additional terms in the Gibbs free energy result from surface or interfacial contributions. As made apparent by the diffraction patterns shown in Figure 3 B, the Mg_0.2_Co_0.2_Ni_0.2_Cu_0.2_Zn_0.2_O HEO material investigated in this work displays very large crystallites, excluding the possibility that surface or interfacial terms play an active role in our case. Thus, we are left with the conclusion that the configurational entropy *S*_config_ = −*R*∑_*i*_χ_*i*_ ln χ_*i*_, where the χ_*i*_ are the mole fractions of the constituents *i*, is the stabilizing contribution to the Gibbs free energy for the rock-salt structure of HEO and is therefore here responsible for the permanence of Cu(II). This last observation can be of extreme importance, as it paves the way for a novel strategy for stabilization of materials with elements in exotic and/or unstable oxidation states.

A final comment concerns the possibility of using the Mg_0.2_Co_0.2_Ni_0.2_Cu_0.2_Zn_0.2_O HEO material as a real catalyst for the CO oxidation reaction. The above results and literature data^10^ show that the HEO is active at temperatures that are well above room temperature. On the other hand, as already mentioned above, oxide catalysts for the CO oxidation reaction, such as Co_3_O_4_, are inactivated by moisture and therefore need to be activated before the reaction. We did not observe any deactivation of the HEO, and we could perform the reaction directly on the as-prepared powder without any preliminary treatment. This indicates that the HEO is resistant toward contamination by moisture. In addition, it should be noted that the large crystals formed by the HEO material used in the present investigation limit the surface area to relatively small values. The possibility of preparing the HEO in the form of nanoparticles is currently under investigation by our group as a starting basis for a complete investigation of the catalytic performance of this material, aiming at lowering the working temperatures.

## Experimental Methods

*Synthesis and Characterization*. Crystalline Mg_0.2_Co_0.2_Ni_0.2_Cu_0.2_Zn_0.2_O was prepared by a sol–gel route starting from the metal nitrates. All of the reagents were purchased at analytical grade from Sigma-Aldrich and used without further purification. The nitrates were dissolved in water, and then citric acid was added (1:1 molar ratio). The reaction mixture was stirred for 12 h at 80 °C and then dried in an oven at 120 °C for 2 h. The resulting powder was then ground with an agate mortar and pestle, calcined for 2 h at 900 °C, and then quenched to room temperature in air. The chemical purity and phase purity were then checked by PXRD.

*Powder X-ray Diffraction Analysis*. Gently ground powders of Mg_0.2_Co_0.2_Ni_0.2_Cu_0.2_Zn_0.2_O were deposited in the 2 mm deep hollow of a zero-background plate (a properly misoriented quartz monocrystal). Diffraction experiments were performed using Cu Kα radiation (λ = 1.5418 Å) on a vertical-scan Bruker AXS D8 Advance diffractometer in θ:θ mode, equipped with a Goebel mirror, a Bruker Lynxeye linear position-sensitive detector, and the following optics: primary and secondary Soller slits, 2.3° and 2.5°, respectively; divergence slit, 0.1°; receiving slit, 2.82°. The generator settings were 40 kV and 40 mA. The nominal resolution for the present setup is 0.08° 2θ (fwhm of the α_1_ component) for the LaB_6_ peak at about 21.3° (2θ). The accurate diffraction patterns of Mg_0.2_Co_0.2_Ni_0.2_Cu_0.2_Zn_0.2_O at room temperature before and after the reaction were acquired in the 10–105° and 10–90° 2θ ranges, respectively, with Δ(2θ) = 0.02° and an exposure time of 2 s/step. Further details on the Le Bail and Rietveld refinements are provided in the Supporting Information.

*XAS Experiment*. For the XAS experiment, a small amount of the Mg_0.2_Co_0.2_Ni_0.2_Cu_0.2_Zn_0.2_O material (ca. 5 mg) in the form of loose powder was hand-pressed on the sample holder of the reaction cell of the APE beamline at the ELETTRA synchrotron radiation facility. The sample holder was fixed with screws onto the titanium base of the cell, which was floating from ground and connected with a coaxial cable. In this geometry, the X-ray beam passes through the membrane and the gas layer and then hits the sample and generates the secondary emission, which is collected by a picoammeter connected to the sample and measuring the drain current. All of the measurements were performed with the sample kept grounded through the picoammeter and a positive bias voltage of 40 V applied to the membrane. The cell was mounted in the UHV chamber of the APE-HE beamline coaxially with the X-ray beam. The reaction cell was mounted on an *x*–*y* table that allowed its movement in the plane perpendicular to the incident beam with 5 μm vectorial precision. This allowed the alignment of the membrane onto the beam. The sample surface, inside the cell, sat at the focal point of the beamline.^23^ The measurements were performed at the Co, Ni, and Cu L_2,3_ edges. Surface sensitivity was obtained by collecting the XAS spectra in total electron yield mode: the estimated probed depth was ca. 3–4 nm.^24^ To ensure maximum gas purity, especially concerning water and carbon oxides, the He carrier gas was passed through a liquid N_2_ trap before entering the cell. The spectra at all of the edges were background-subtracted by fitting the pre-edge with a straight line and then normalized to unit absorption after the L_3_ edge, although it was explicitly stated that non-normalized spectra are shown. The experiments were conducted in flowing He (50 standard cubic centimeters per minute, SCCM), either pure or with the addition of CO (2 SCCM), O_2_ (2 SCCM), or a stoichiometric CO + O_2_ mixture (2 + 1 SCCM, respectively). All of the gases were supplied by Linde, with a purity of at least 99.999%. The CO_2_ concentration in the exhaust pipeline of the APE operando cell was measured by means of a nondispersive infrared CO_2_ sensor (Gravity, Dfrobot SEN0219). The sensor was completely embedded in the gas flowing out of the reaction cell, and its response was converted to CO_2_ concentration by means of a National Instrument data acquisition interface after calibration with a standard (Linde, 99.999%). The sensor output, transformed to the fraction of converted CO, is shown in Figure S2, and it is in good agreement with previous reports.^10^ Multiplet calculations were performed by means of the XTM4XAS program,^25^ including crystal field, charge transfer, and spin–orbit coupling effects.

## Supporting Information Available

Comparison of the experimental spectra with theoretical calculations (Figure S1), CO oxidation rate in the temperature range of interest (Figure S2), Cu L_2,3_-edge spectra of the high-entropy oxide in the CO + O_2_ mixture at temperatures below the start of the CO oxidation reaction (Figure S3), determination of the Cu(I) fraction (Figure S4 and Table S1), discussion of the choice of soft-XAS as a mechanistic probe for the CO oxidation over the Mg_0.2_Co_0.2_Ni_0.2_Cu_0.2_Zn_0.2_O HEO, and details of the powder X-ray diffraction analysis (PDF)
