# Supplementary material for: Stabilization by Configurational Entropy of the Cu(II) Active Site during CO Oxidation on Mg0.2Co0.2Ni0.2Cu0.2Zn0.2O
Source: J Phys Chem Lett. 2020 Apr 20;11(9):3589–93. doi: 10.1021/acs.jpclett.0c00602 (PMC8007101; doi:10.1021/acs.jpclett.0c00602)
Supplement: Supplementary file 1 — jz0c00602_si_001.pdf [file jz0c00602_si_001.pdf]

Supporting Information

Stabilization by Configurational Entropy of the Cu(II) Active Site during CO Oxidation on $Mg_{0.2}Co_{0.2}Ni_{0.2}Cu_{0.2}Zn_{0.2}O$

Martina Fracchia[1], Paolo Ghigna[1,2], Tommaso Pozzi[1], Umberto Anselmi Tamburini[1,2], Valentina Colombo[2,3], Luca Braglia[4], Piero Torelli[4]*

[1]Dipartimento di Chimica, Università di Pavia, V.le Taramelli 13, I-27100, Pavia, Italy

[2]INSTM, Consorzio Interuniversitario per la Scienza e Tecnologia dei Materiali, Via Giusti 9, 50121 Firenze, Italy

[3]Dipartimento di Chimica, Università degli Studi di Milano, Via Golgi 19, I-20133, Milano, Italy.

[4]CNR- Istituto Officina dei Materiali, TASC, Trieste, Italia

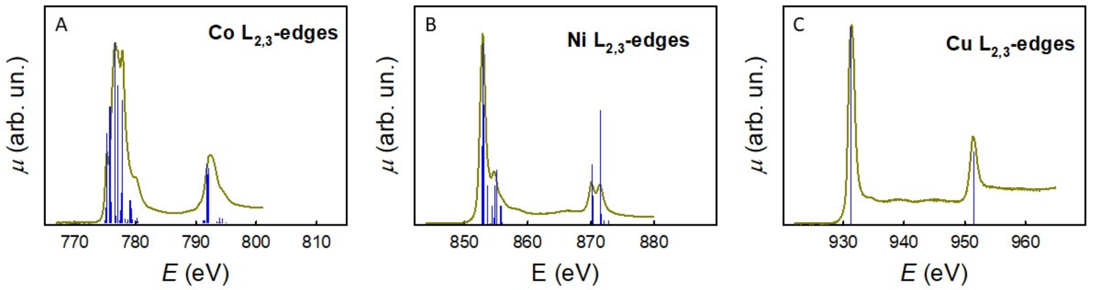

Figure S1 – Comparison of the experimental Co (panel A), Ni (panel B) and Cu (panel C) $L_{2,3}$-edge spectra with multiplet calculations performed with the XTM4XAS program (blue bars). The experimental spectra are at 100 °C in He in all cases. The local symmetry for calculations is assumed to be O_h in all the three cases.

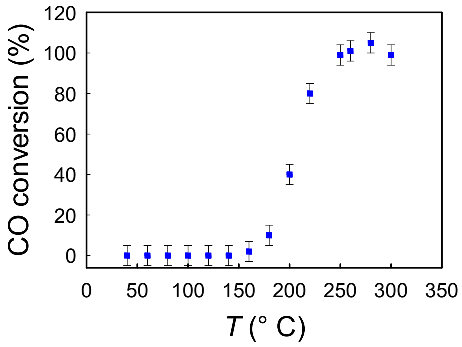

Figure S2 – CO oxidation activity of the $Mg_{0.2}Co_{0.2}Ni_{0.2}Cu_{0.2}Zn_{0.2}O$ high entropy oxide.

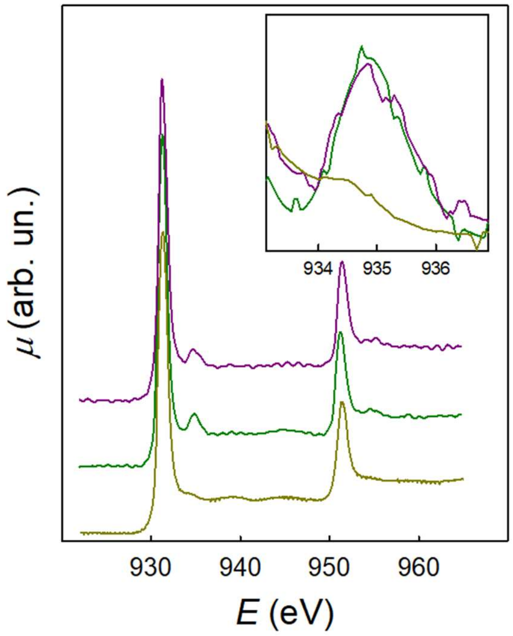

Figure S3 – Cu $L_{2,3}$-edges spectra of the $Mg_{0.2}Co_{0.2}Ni_{0.2}Cu_{0.2}Zn_{0.2}O$ high entropy oxide in the $CO+O_2$ mixture at temperatures below the starting of the CO oxidation reaction. The inset shows the region of the Cu(I) peak on an enlarged scale.

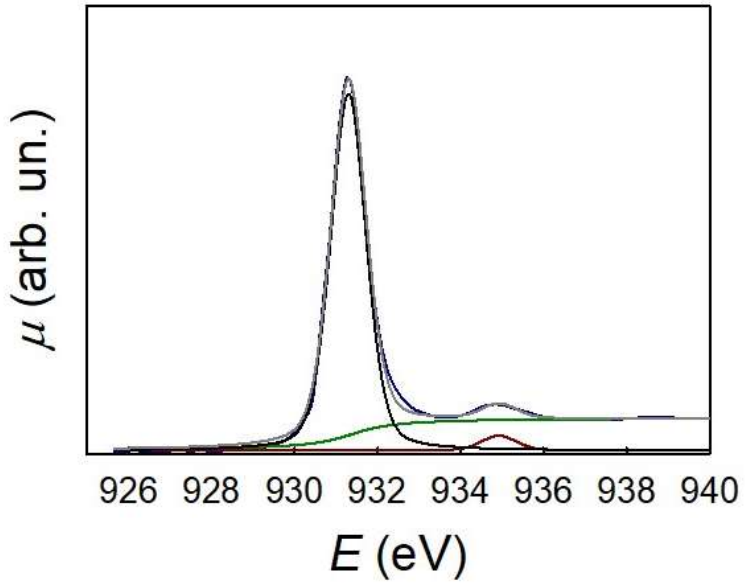

Figure S4 – Cu L_3-edge spectrum of the $Mg_{0.2}Co_{0.2}Ni_{0.2}Cu_{0.2}Zn_{0.2}O$ high entropy oxide in the $CO+O_2$ mixture at 250 °C (dark blue line, data from Fig. 1A), fitted with an arctangent function (dark green) and two pseudo Voight peaks for Cu(II) and Cu(I) (black and dark red lines, respectively). The fit is shown as a grey line. The areas of the two peaks allows to determine the fraction of Cu(I). First, these areas need to be normalised by the cross sections of Cu(I) and Cu(II) in the rock salt structure. The first one can be easily obtained from the spectrum of the starting HEO (100 °C in He, dark yellow line in Fig. 1A), performing a similar fitting to that shown in the present figure with just one pseudo Voight. As the second one is inaccessible, as an approximation we use the spectrum of Cu_2O (dark red line in Fig. 1B). After the normalisation, the data shown in Tab. S1 are obtained. A further warning concerns the normalisation of the Cu_2O L_3-edge spectrum, which is difficult due to the presence of relevant structures after the edge. As a consequence, the data displayed in Tab. S1 should be considered as semiquantitative.

Cu(I) fraction (atomic percent)	Conditions
3 %	250 °C, CO + O_2
8 %	250 °C

Tab S1. Fraction of Cu(I) in the $Mg_{0.2}Co_{0.2}Ni_{0.2}Cu_{0.2}Zn_{0.2}O$ high entropy oxide in different conditions, obtained as explained above.

Choice of soft-XAS as a mechanistic tool.

Hard X-ray techniques represent a wide portfolio of invaluable techniques for the characterization of materials, where hard X-ray diffraction and spectroscopies are presently the working horses for both ex- and in-situ investigations. For instance, XAS in the hard X-ray regime is an irreplaceable tool in the investigation of local atomic and electronic structures of materials, and operando XAS experiments with hard X-rays are well established and almost every experimental condition can be reached to simulate realistic reaction environments. However, considering a surface reaction taking place on a 3*d* transition metal compound, hard X-ray XAS suffers of a series of limitation.

First, the 3*d* transition metal K-edges in the 4.5-10 keV range, are associated with the 1*s* initial state. According to the $\Delta\ell=\pm1$ selection rule, the final states have *p* character, and the 3*d* valence states can be accessed via quadrupolar transitions only, whose intensity is generally very small, if local symmetry related effects, allowing mixing of *d* and *p* states, do not occur. Secondly, the penetration depth of X-rays in this energy range can be of several microns, and therefore surface effects and/or reactions can be well beyond the sensitivity limit of the technique, which is, at best, of few atomic %.

In the soft X-ray regime, on the contrary, at the $L_{2,3}$ edges in the 400 – 1200 eV energy range, the initial states have $2p$ character, so that the $3d$ valence states are directly accessible via dipolar transitions.

In addition, the penetration depth of X-rays in this energy range amounts to hundreds of nm in the worst case, and therefore the surface contributions to the spectra is enhanced.

This is particularly true when soft-XAS is operated in the Total Electron Yield (TEY) detection method. Indeed, in the TEY mode, the absorption coefficient is measured by detecting all the electrons (photoelectrons, Auger electrons, secondary electrons) emitted from the sample after photoabsorption are collected. In the TEY the probed depth of sample is not limited by the penetration depth of the incoming X-rays, but by the escape depth of the electrons. Such electrons emitted in the soft X-ray regime have moderate kinetic energies, and their escape depth is very small, of the order of few nanometers. This means that only very few atomic layers below the surface can be probed.

The total electron yield detection mode can be in principle implemented also in the hard X-ray regime. However, synchrotron radiation beamlines working at high energy are usually not equipped with this kind of detection.

X-ray Powder Diffraction Analysis

Powder X-ray Diffraction analysis were performed with TOPAS-Academic 6.[1] The peak shapes were described with the fundamental parameters approach[2]. The background was modelled by a Chebyshev polynomial function. The thermal effect was simulated by using two refinable isotropic Debye-Waller parameters for the oxygen ion and the cations, respectively.

On the as-synthesized $Mg_{0.2}Co_{0.2}Ni_{0.2}Cu_{0.2}Zn_{0.2}O$ sample, Le Bail refinement was carried out in order to determine the exact cell parameter (a for the cubic *Fm-3m* cell), background and profile parameters to be used in the Rietveld refinement. The final Le Bail and Rietveld refinement plots are supplied in Figure S5 and S6, respectively. On the treated sample, Le Bail refinements were

performed in the cubic *Fm*$\bar{3}$*m*; trigonal *R*$\bar{3}$*m* and tetragonal *I4/mmm* space groups. The plots are reported in Figure S7 and S8 and S9, respectively.

In the case of the as-synthetized sample of composition $Mg_{0.2}Co_{0.2}Ni_{0.2}Cu_{0.2}Zn_{0.2}O$, a very good Rietveld refinement was obtained assuming the random distribution of the cations on one of the *fcc* sublattices. This is also accompanied by a reasonably low Debye-Waller factor ($B_{iso}(M^{2+})$ = 0.46(4) Å; see Figure S6), which is a good sign of very low contribution (*or any*) of a deformation term on the thermal parameters. On the contrary, the effect of lattice deformation on the thermal parameter has been reported for High Entropy Alloys (HEA), where a lattice deformation gives rise to a rapid decrease of the intensities of the Bragg peaks at high angles and an additional term in the Debye-Waller factor need to be taken into consideration for a good Rietveld refinement.[3]

To exclude a possible stoichiometric deviation from the ideal distribution (with each of the M^{2+} cation with occupancy factor 0.2) another observation can be done on the relative intensities of the Bragg peaks. Indeed, for an ideal rock-salt structure with random distribution of the cations, the relative intensity of the calculated Bragg peaks with Miller indices (111) and (200) is I_{111}/I_{200} = 0.67. On our sample, the experimental pattern shows a ratio equal to 0.70, which can be considered in good agreement with the expected stoichiometry from the synthesis procedure and with the good agreement obtained in the Rietveld refinement.

To understand and describe the origin of the different intensity ratio and peak widening found in the thermal treated sample of this work, we have calculated two possible structural distortion of the cubic unit cell, namely a rhombohedral and a tetragonal one, towards the two non-isomorphic subgroups *R*$\bar{3}$*m* and *I4/mmm* with a close look to the splitting of the $(200)_c$; $(220)_c$ and $(311)_c$, which are those suffering of line broadening. However, the evolution of the cubic crystal structure seems to be more probable toward a tetragonal distortion rather than to a rhombohedral one, because the splitting of the peaks expected for the *I4/mmm* distortion very well matches with the observed widening of the $(200)_c$; $(220)_c$ and $(311)_c$ reflections. More in detail, the rhombohedral distortion (*R*$\bar{3}$*m*; a, 5.9904(1); c,

7.3596(3) Å) foresee a splitting of the $(111)_c$ reflection into (003)+(021) with the $(200)_c$ peak that remain unchanged; while in the tetragonal one (*I4/mmm*; a, 2.9919(2); c, 4.2520(4) Å) the $(111)_c$ peak remain unchanged, and the observed broadened peaks are indeed split into (110)+(002); (020)+(112); (211)+(013) for the $(200)_c$; $(220)_c$ and $(311)_c$ reflections.

Figures S7, S8 and S9 report the Le Bail refinements for the cubic phase; the rhombohedral and the tetragonal distortions, respectively. Even if the agreement in the refinement is not fully satisfying, the best result is, as expected, obtained with the tetragonal distortion, which well describes the line broadening with peaks splitting exactly on the observed widened peaks. It should be noted here that another effect of distortion maybe need to be taken into account to reach a better agreement of the refinement, indeed, a detailed study is ongoing in our group. As already reported by Berardan *et al.*[4] the competition between the local deformation given by the Jahn-Teller effect of the copper ions in the octahedral environment and the other cations, which prefer the regular octahedral environment, induces both a slight tetragonal distortion of the unit cell, which give rise to the splitting and broadening of the $(200)_c$; $(220)_c$ and $(311)_c$ Bragg peaks, and a possible second lattice deformation, that leads to the decrease of the intensity of the same peaks.

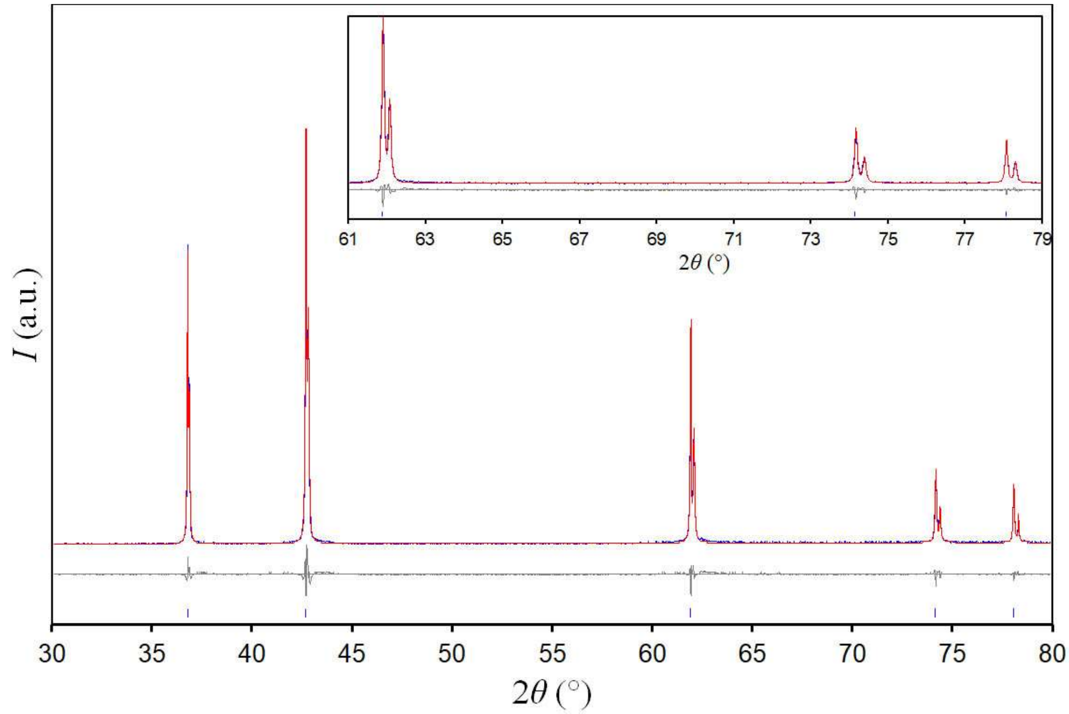

Figure S5. Le Bail refinement plot of the as-synthetized $Mg_{0.2}Co_{0.2}Ni_{0.2}Cu_{0.2}Zn_{0.2}O$ HEO material in the rock-salt *Fm-3m* space group. Blue trace and red trace represent experimental and calculated diffractograms, respectively. Grey line, represents the difference between experimental and calculated profiles. Blue tick marks are the calculated peaks positions. The inset shows an enlargement of the high angle region. R_p and R_{wp}, 0.1032 and 0.1398 respectively, for 3751 data in the 30-105 2θ range.

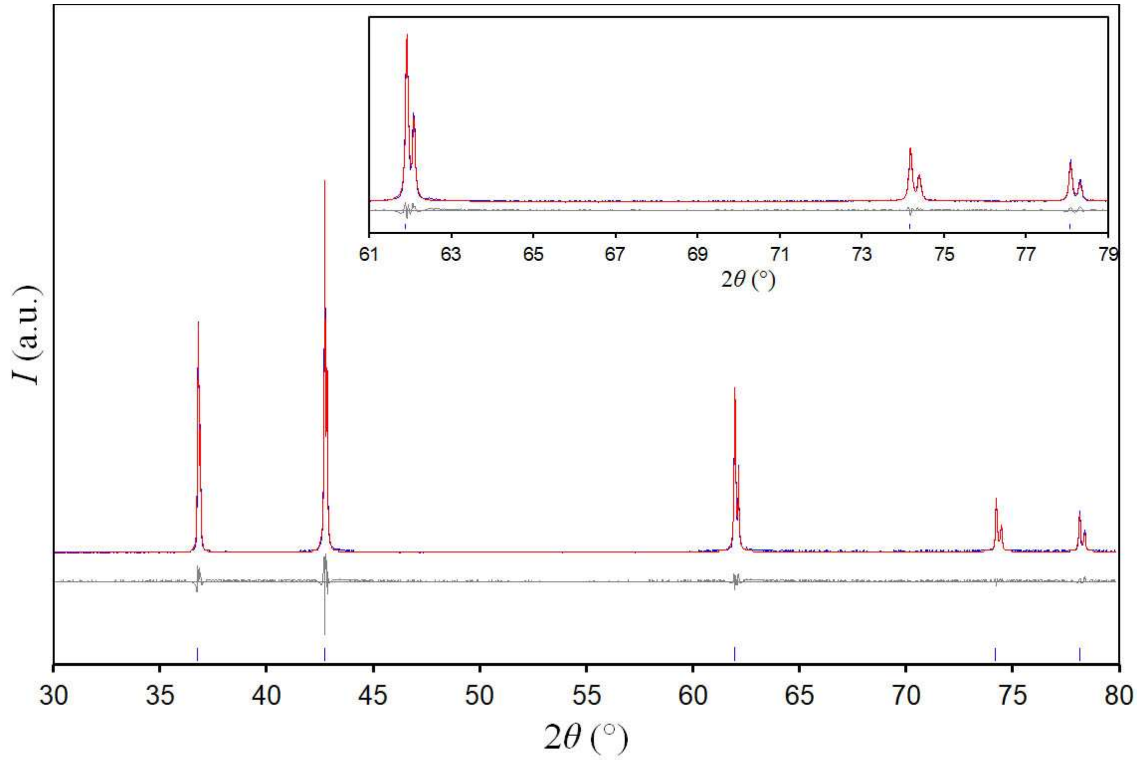

Figure S6. Rietveld refinement plot of the as-synthetized $Mg_{0.2}Co_{0.2}Ni_{0.2}Cu_{0.2}Zn_{0.2}O$ HEO material. Structure solution in the rock-salt *Fm-3m* space group (*a*, 4.2366(5) Å). Blue trace and red trace represent experimental and calculated diffractograms, respectively. Grey line, represents the difference between experimental and calculated profiles. Blue tick marks are the calculated peaks positions. The inset shows an enlargement of the high angle region. R_p; R_{wp} and R_{Bragg}, 0.1109; 0.1480 and 1.956, respectively, for 3751 data in the 30-105 2θ range. The isotropic Debye-Waller factor was refined to $B_{iso}(M^{2+})$ = 0.46(4) Å and, similarly, to $B_{iso}(O^{2-})$ = 0.46(8) Å.

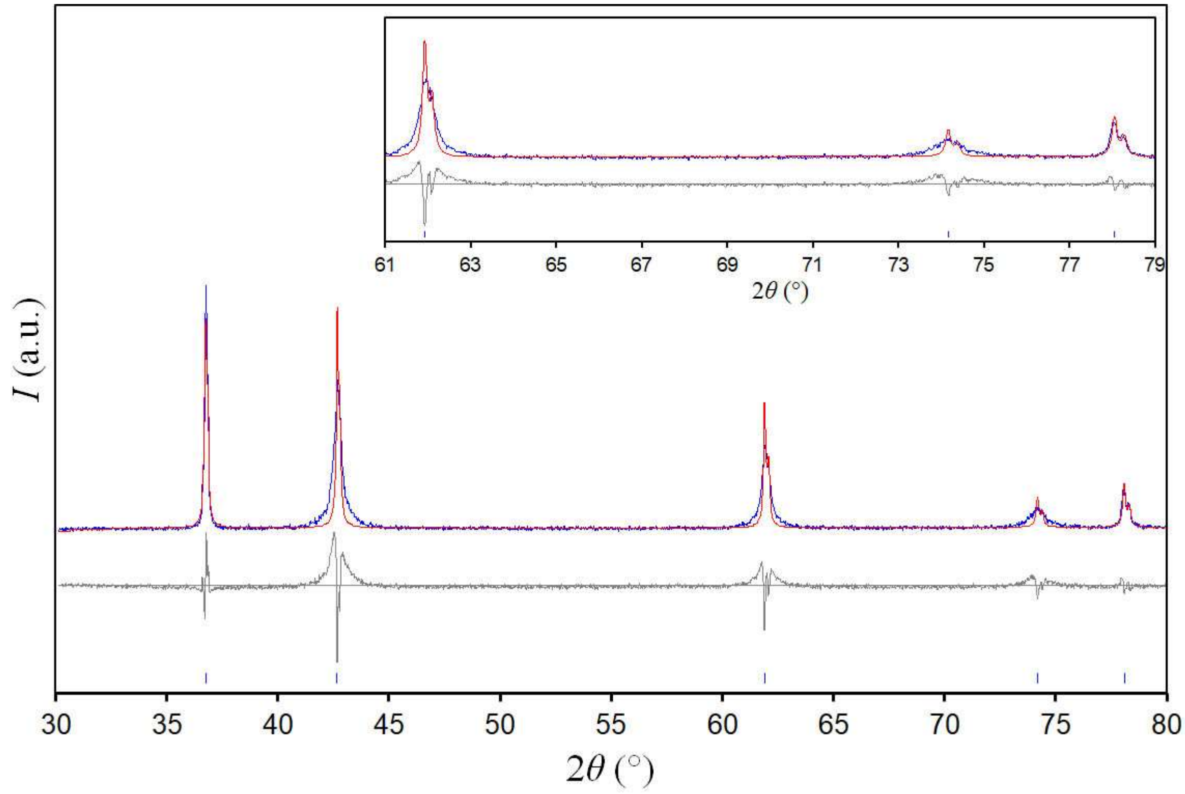

Figure S7. Le Bail refinement plot of the $Mg_{0.2}Co_{0.2}Ni_{0.2}Cu_{0.2}Zn_{0.2}O$ HEO material after the thermal treatments described in this work in the rock-salt *Fm-3m* space group (a, 4.2366(5) Å). Blue trace and red trace represent experimental and calculated diffractograms, respectively. Grey line, represents the difference between experimental and calculated profiles. Blue tick marks are the calculated peaks positions. The inset shows an enlargement of the high angle region. R_p and R_{wp}, 0.1448 and 0.2014 respectively, for 3001 data in the 30-90 2θ range.

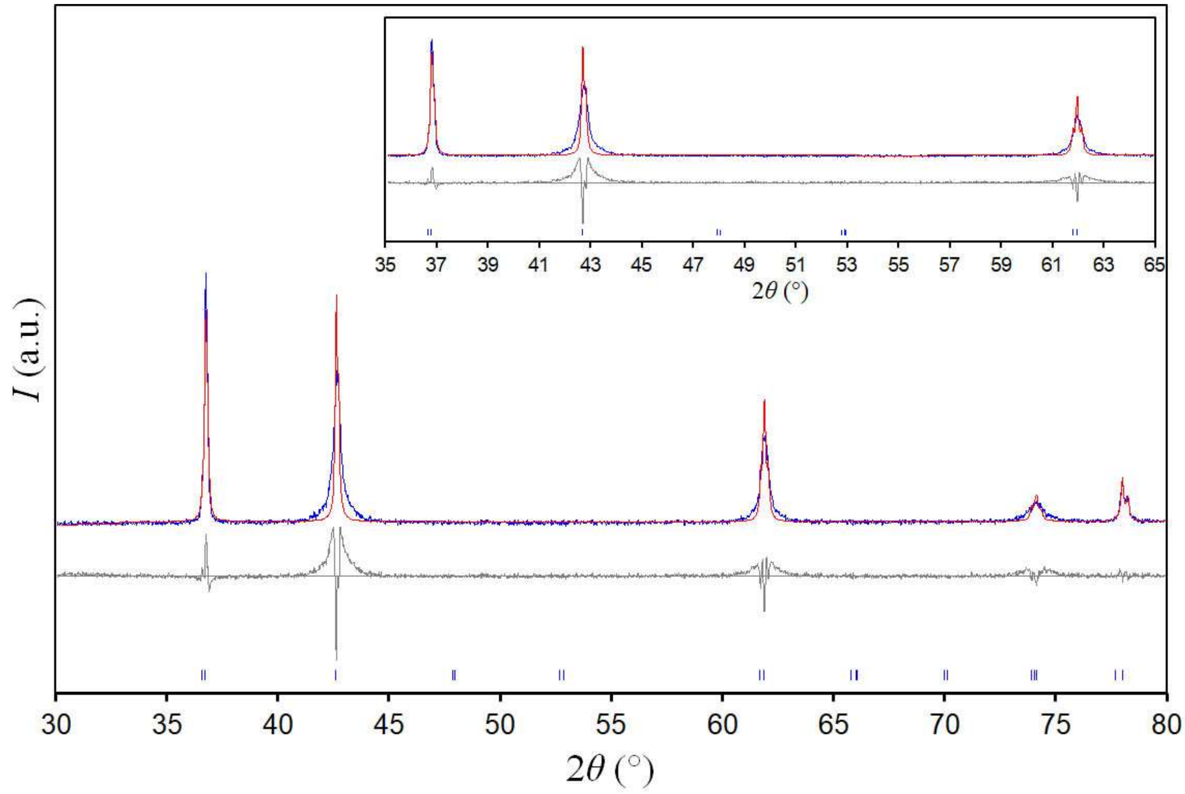

Figure S8. Le Bail refinement plot of the $Mg_{0.2}Co_{0.2}Ni_{0.2}Cu_{0.2}Zn_{0.2}O$ HEO material, after the thermal treatments described in this work, for the rhomobohedral distortion, space group *R-3m* (a, 5.9904(1); c, 7.3596(3) Å). Blue trace and red trace represent experimental and calculated diffractograms, respectively. Grey line, represents the difference between experimental and calculated profiles. Blue tick marks are the calculated peaks positions. The inset shows an enlargement of the first bragg peaks. R_p and R_{wp}, 0.1387 and 0.1979 respectively, for 3001 data in the 30-90 2θ range.

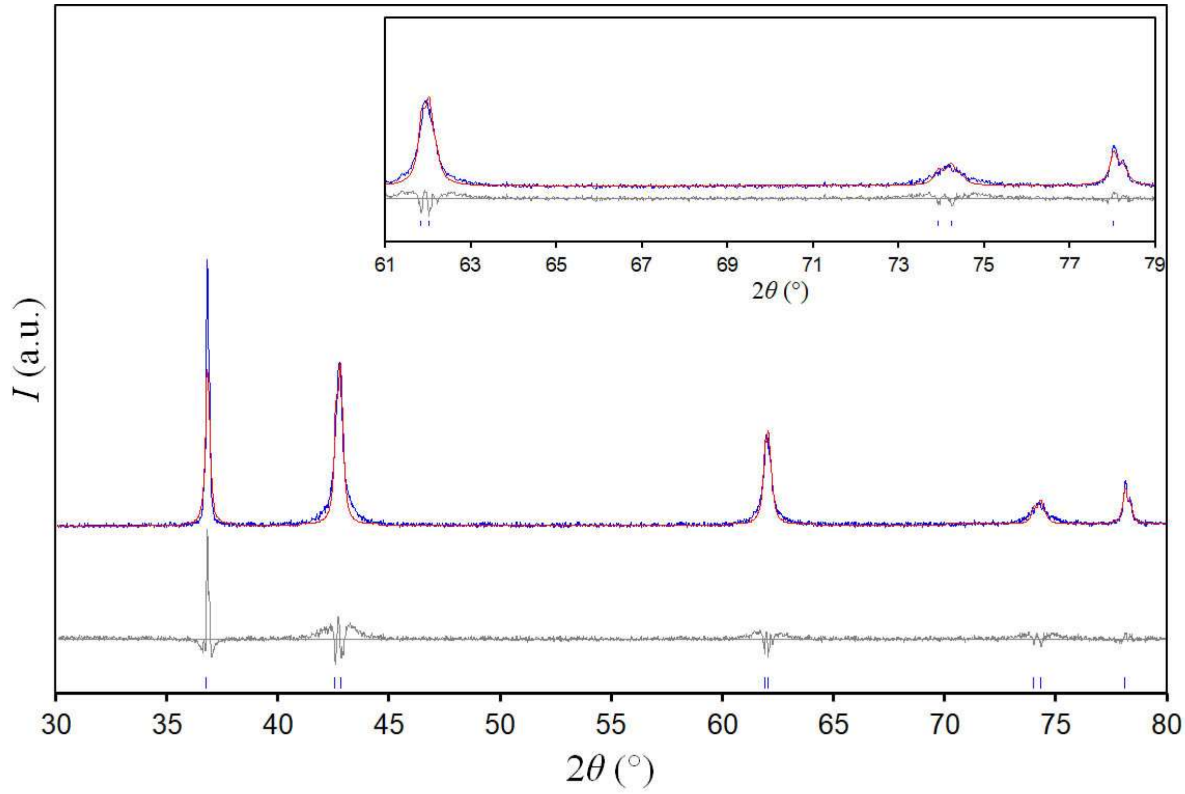

Figure S9. Le Bail refinement plot of the $Mg_{0.2}Co_{0.2}Ni_{0.2}Cu_{0.2}Zn_{0.2}O$ HEO material, after the thermal treatments described in this work, for tetetragonal distortion, space group *I4/mmm* (a, 2.9919(2); c, 4.2520(4) Å). Blue trace and red trace represent experimental and calculated diffractograms, respectively. Grey line, represents the difference between experimental and calculated profiles. Blue tick marks are the calculated peaks positions. The inset shows an enlargement of the high angle region. R_p and R_{wp}, 0.1098 and 0.1457 respectively, for 3001 data in the 30-90 2θ range.

References

[1] Coelho, A.A.; TOPAS and TOPAS-Academic: an optimization program integrating computer algebra and crystallographic objects written in C++. *J. Appl. Cryst.* **2018**, 51, 210-218.

[2] Cheary, R.W.; Coelho, A.A. A fundamental parameters approach to X-ray line-profile fitting *J. Appl. Cryst.* **1992**, 25, 109.

[3] Yeh, J.-W.; Chang, S.-Y.; Hong, Y.-D.; Chen, S.-K.; Lin, S.-J. Anomalous decrease in X-ray diffraction intensities of Cu-Ni-Al-Co-Cr-Fe-Si alloy systems with multiprincipal elements, *Mat. Chem. Phys.* **2007**, 103, 41.

[4] Berardan, D.; Meena, A.K.; Franger, S.; Herrero, C.; Dragoe, N. Controlled Jahn-Teller distortion in (MgCoNiCuZn)O-based high entropy oxides *J. Alloys Compd.* **2017**, 704, 693-700
